# Accelerated Decline in Sense of Purpose in Life Years Prior to Dementia Diagnosis

**DOI:** 10.1093/geroni/igaa057.901

**Published:** 2020-12-16

**Authors:** Nathan Lewis, Jonathan Rush, Patrick Hill

**Affiliations:** 1 University of Victoria, Victoria, British Columbia, Canada; 2 Washington University in St. Louis, Saint Louis, Missouri, United States

## Abstract

Research has pointed to sense of purpose in life as an important individual difference that promotes successful aging, predicting greater psychological wellbeing, physical functioning, and cognitive health in older adulthood. Despite such benefits, it is unclear how major life challenges such as the onset of cognitive impairment may impact the sense of purpose of older adults. Building on this, the present study examined long-term change in sense of purpose in a longitudinal sample of older adults who would later develop dementia. Data were from 341 participants without dementia at study intake who were subsequently diagnosed with dementia during the follow-up period (Mage = 81.92 years at intake, Mage = 89.19 years at diagnosis, 72.72% female). Participants completed annual assessments of purpose in life up to 17 years prior to dementia diagnosis (M = 6.99 assessments prior to diagnosis). Piecewise growth modeling examined change in sense of purpose in life with time structured as years to diagnosis. Results suggested a period of accelerated decline distinct from normative age-related change beginning around 5.60 years prior to dementia onset. Discussion will focus on two potential explanations: that perceiving declines in cognition may lead to diminished sense of purpose in life or that underlying changes in the brain may impact both cognition and sense of purpose resulting in concurrent decline over time.

